# A continuum study of layer analysis for single species ion transport inside double-layered graphene sheets with various separations

**DOI:** 10.1038/s41598-019-48101-8

**Published:** 2019-08-12

**Authors:** Yue Chan, Muhammad Saeed, Shern-Long Lee, Jonathan J. Wylie

**Affiliations:** 1 Institute for Advanced Study, Shenzhen University, Nanshan District Shenzhen, Guangdong, 518060 P.R. China; 2 College of Nuclear Science and Engineering, East China University of Technology, Nanchang, 330013 P.R. China; 3 Department of Mathematics, City University of Hong Kong, Tat Chee Avenue, Kowloon, Hong Kong SAR

**Keywords:** Applied mathematics, Mechanical and structural properties and devices

## Abstract

We investigate the formation of thin ionic layers driven by electro-osmotic forces, that are commonly found in micro- and nano-channels. Recently, multi-layers have been reported in the literature. However, the relation between classical Debye layers and multi-layers, which is a practically and fundamentally important question, was previously unexplained. Here, we fill this gap by using a continuum approach to investigate the flow of lithium ions inside double-layered graphene sheets. Fluid flow, charge conductivity and thermal stability will be investigated. We show that the separation and strength of forces between the sheets, the external electric field and thermal effects determine the topology of the ionic layers between the graphene sheets.

## Introduction

Double layers refer to the thin layers of charges, that form near the surface of objects, where the thickness of layers can be characterized by the Debye length. Such layers commonly occur in large surface area to volume ratio systems at the micro- and nano-scale accounting for some important phenomena including electrochemical behavior of electrodes^1^. Asymptotic matching is a common technique to solve the boundary layer problem for double layers^1,2^. Recently, multi-layers that are comprised of free ions, occupy the entire cavity were shown to occur at the nanoscale^3–5^. However, the relationship between the classical double layers and multi-layers is missing. Here, we adopt the Poisson-Nernst-Planck equation in conjunction with the mean field theory to investigate the ion transport inside nanomaterials including double-layered graphene sheets. Graphene sheets have a huge potential to be used as anodes for high capacity lithium-ion batteries. Graphene-related materials^6–8^ and graphene-based composite materials^9–11^ are also found to enhance charge storage of lithium-ion batteries.

Graphene sheets possess superior mechanical, electronic and magnetic properties to form promising materials for modern applications^12^. Due to these merits, atomistic models have been used to simulate lithium ion transport between two graphene sheets^13^. Density functional theory has also been employed to investigate the bonding and magnetic properties of metal atoms encapsulated inside graphite^14^. However, these first principle calculations are limited when analyzing the existence of multi-layers due to the heavy computational time needed for such methods. Therefore, a continuum approach turns out to be a computationally effective way to describe the ion transport in mesoscopic systems. Chan and Hill^15^ developed an empirical model to study the lithium storage and diffusion times between graphene. Diffusion and transport models^16,17^ have also been derived for depicting charge transport in lithium-ion batteries, where electro-chemical factors and boundary conditions for different components can be carefully addressed. Multi-scale modeling^2^ taking into account the merits of both atomistic and continuum approaches has also be used to tackle ion storage problems for lithium-ion batteries^18^. Shi *et al*.^18^ predicted the path-independent properties of lithium ion batteries in multiple spatial and temporal scales.

Amongst those continuum approaches, the Poisson-Nernst-Planck (PNP) equation^19–21^ has proven to be extremely useful in scrutinizing ion transport inside systems with larger length scales. Ernesto *et al*.^16^ adopted the PNP equation and took into account Faraday’s and Ohm’s laws for both the electrode and electrolyte to derive important quantities such as the cell voltage, and the electrode and electrolyte potentials for lithium-ion batteries^16^. Their research provides a vital theoretical background to determine certain electrical properties for lithium-ion batteries, which is crucial to supplement experimental efforts. More importantly, analytic and asymptotic solutions^20,22^ have been sought for the PNP equation for some simple geometries. However, the above continuum approaches using the PNP equation fail to address ion transport at the nanoscale as the molecular effects are significant in a porous cavity. Meng *et al*.^3^ employed the PNP equations in conjunction with a classical density functional theory to describe lithium ion flow and discover multi-layers at the nanoscale. The present authors have also successfully incorporated molecular and steric effects into the PNP equation to investigate particle-laden flow problems at the nanoscale^4,5^, where the force fields are approximated by the mean field theory^23,24^ and the steric effects are computed using the number density derived from the PNP equation. Mean field theory has been successfully employed in condensed matter physics^25^, ultra-filtration using carbon nanotube membranes^26,27^, axial buckling of nanopeapods^28^, nano drug delivery^29^ and nanoshuttle memory devices^30^.

In this paper, we show that the combination of the PNP equation and the mean field theory is a computationally effective method for describing the ionic flow inside regular nanomaterials, and can predict the double and multi-layers inside double-layered graphene. This provide a theoretical background for the establishment of the relation between different types of layers. In the following sections, numerical methods for solving the layering problem will be derived. These methods will be used to derive criteria for the occurrence and the properties of double and multi-layers. The transition between double and multi-layers will also be discussed. Some approximate analytical treatments are also provided to support our argument.

## Methods

In this section, we present a general theoretical framework for ion transport inside nanomaterials such as double-layered graphene. Such methods have successfully been used in^4,5^. Both the approximate theoretical treatment and the numerical methods for the present problem will be discussed later, and the extension to other devices such as lithium-ion batteries, and photovoltaic and fuel cells can be found in^16,17^, where addition conditions such as the neutrality is assumed and the dynamics for each components is taken into account.

### General theory

Suppose the flux of (any) ions inside a nanomaterial is driven by the diffusion generated by the concentration gradient, advection by the flow field, and molecular interactions among ions and between ions and the host material, and is given by

1 $${\bf{J}}=-D\nabla c+c{\bf{u}}+\frac{D}{{k}_{B}T}c{\bf{F}},$$

where *c*, **u**, *D*, *k*_*B*_, *T* and **F** denote the ion concentration, the fluid velocity, the diffusion coefficient, the Boltzmann constant, the temperature and the total intrinsic force between ions and between the ion and the host nanomaterial, respectively. The conservation of mass inside the host material gives the Nernst-Planck-type equation^1^

2 $$\frac{\partial c}{\partial t}=-\nabla \cdot {\bf{J}}=\nabla \cdot \{D\nabla c-c{\bf{u}}-\frac{D}{{k}_{B}T}c{\bf{F}}\}.$$

For constant *D* and *T*, Eq. (2) reduces to

3 $$\frac{\partial c}{\partial t}=D{\nabla }^{2}c-\nabla \cdot (c{\bf{u}})-\frac{D}{{k}_{B}T}\{{\bf{F}}\cdot \nabla c+c\nabla \cdot {\bf{F}}\}.$$

The flow field **u** can be described by the Navier-Stokes equation for a Newtonian fluid and the incompressibility condition subject to the external electric field, **E**,

4 $$\begin{array}{rcl}\rho \frac{\partial {\bf{u}}}{\partial t}+\rho {\bf{u}}\cdot \nabla {\bf{u}} & = & -\nabla p+\eta {\nabla }^{2}{\bf{u}}+{\rho }_{E}{\bf{E}},\\ \nabla \cdot {\bf{u}} & = & 0,\end{array}$$

where *ρ*, *η* and *ρ*_*E*_ denote the fluid density, viscosity and charge density, respectively. Furthermore, the charge density can be derived from Poisson’s equation, which is given by

5 $${\rho }_{E}=-\varepsilon {\nabla }^{2}\varphi ,$$

where *ε* and *φ* denote the electrical permittivity and the potential induced by the ions. We assume that the interaction of ions is subject to non-bonded electrical and van der Waals forces, which can be modeled by

6 $$V({r}_{ij})=\frac{{q}_{i}{q}_{j}}{{r}_{ij}}+4{\varepsilon }_{ij}[{(\frac{{\sigma }_{ij}}{{r}_{ij}})}^{12}-{(\frac{{\sigma }_{ij}}{{r}_{ij}})}^{6}],$$

where *r*_*ij*_, *q*_*i*_, *ε*_*ij*_ and *σ*_*ij*_ denote the atomic distance between the *i*-th and *j*-th atoms, charge for *i*-th ion, the potential well depth between *i* and *j* atoms and the van der Waals diameter between *i*-th and *j*-th atoms. In addition, *σ*_*ij*_ = (*σ*_*i*_ + *σ*_*j*_)/2 and ${\varepsilon }_{ij}=\sqrt{{\varepsilon }_{i}{\varepsilon }_{j}}$. Following the account given by Cox *et al*., the total pairwise interaction, *E* between two ions/molecules with surface areas *S*_1_ and *S*_2_ can be approximated by^23,24^

7 $$U=\sum _{i\ne j}V({r}_{ij})\approx {n}_{1}{n}_{2}\int \int V({r}_{ij})d{S}_{1}d{S}_{2},$$

where *η*_1_ and *η*_2_ denote the number density of atoms on the surface *S*_1_ and *S*_2_, respectively. Given *U*, the force field between two molecules can be easily computed by **F** = −▽*U*. We comment that Eqs (3–5) and (7) constitute the major equations for this paper.

### Double-layered graphene

**Figure 1:**
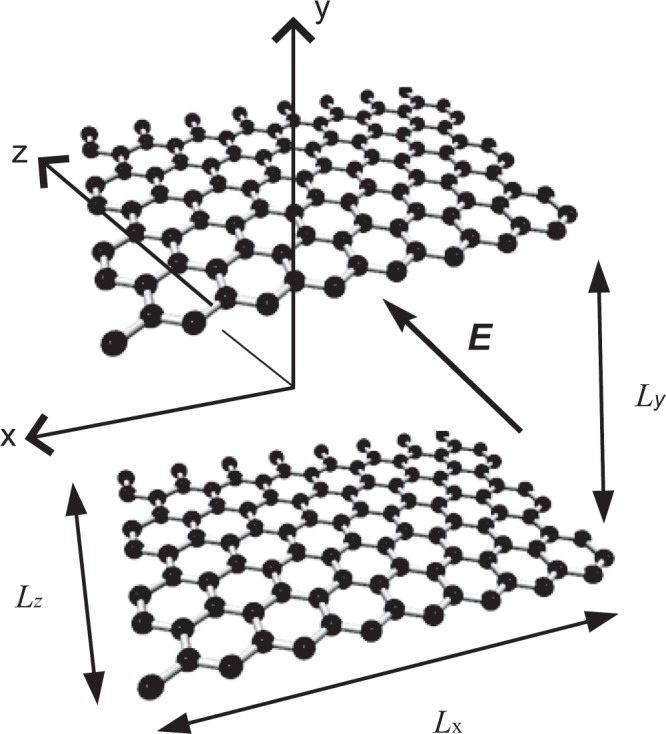
Double-layered graphene sheets, where the coordinate system adopted in this paper and the direction of the external electric field are shown.

Here, we consider the transport of lithium ions inside double-layered graphene, where the coordinate system used in this paper and the direction of the electric field are shown in Fig. 1. To generate tractable numerical results, we only investigate finite systems with separation between graphene sheets, *L*_*y*_ and base area, *L*_*x*_ × *L*_*z*_. But, the voltage difference of 5 V is applied into the system to produce physically realistic results. We also assume the periodic conditions in the *x* and *z* directions, and use Dirichlet condition for fluid flow and ionic concentration in the *y* direction.

The electric field is applied parallel to the graphene sheets in the *z* direction. The *x*-axis is defined to be parallel to the graphene sheets but perpendicular to the field direction, while the *y*-axis is defined to be perpendicular to the graphene sheets (see Fig. 1). We smear the carbon atoms on the surface of graphene so that the interaction between lithium ions and graphene can be described by the 6–12 Lennard-Jones potential^31^. Given that, the total force acting on a lithium ion inside the cavity^5^ is approximated by Eq. (7), this is given by

8 $$\begin{array}{rcl}{F}_{x} & = & H{H}_{x},\\ {F}_{y} & = & \eta \pi (-2A[\frac{1}{{y}^{5}}-\frac{1}{{({L}_{y}-y)}^{5}}]+2B[\frac{1}{{y}^{11}}-\frac{1}{{({L}_{y}-y)}^{11}}])+H{H}_{y},\\ {F}_{z} & = & H{H}_{z}\end{array}$$

where *η*, $A=4{\varepsilon }_{ij}{\sigma }_{ij}^{6}$ and $B=4{\varepsilon }_{ij}{\sigma }_{ij}^{12}$ denote the number density of carbon on the graphene, the attractive and repulsive constants for the lithium ions and carbon, respectively. Unlike the usual PNP formulation at the microscale^32^, ion-ion interactions are significant at the nanoscale. We describe these interactions by **HH**(**r**) at **r**, which is mediated by the average field generated by other ions from **x** positions, where **x** ≠ **r**. By considering both electric and ionic interactions, i.e. Eq. (6) and following the account given by^33^, we model such ion-ion interactions by the integral

9 $${\bf{HH}}({\bf{r}})=-c({\bf{r}})\int \{c({\bf{x}})\nabla (\frac{{B}_{I}}{|{\bf{r}}-{\bf{x}}{|}^{12}}+\frac{{q}_{r}{q}_{x}}{|{\bf{r}}-{\bf{x}}|})\}d{\bf{x}},$$

where *c*(**r**), *c*(**x**), *q*_*r*_, *q*_*x*_, *B*_*I*_ and *d***x** denote the number density of ions at **r**, the number density of ions at **x**, charges at the site *r*, charges at the site *x*, the ion-ion repulsive constant and the volume element for **x**, respectively. Following^33^, cut-off lengths are introduced to obtain convergent results since singularities occur when **x**→**r**. We comment that graphene induces forces on ions only in the *y* direction, whereas steric effects act on the ions from all directions (see Eq. (8)).

Now, the flow field inside the cavity is driven by the external electric field, which is also affected by the feedback of electric field generated by ions and the base voltage (if applicable). We further assume that the flow is sufficiently slow at the nanoscale so that Eq. (4) reduces to Stoke’s flow, from which the steady solution can be obtained by solving

10 $${\eta }_{v}{\nabla }^{2}u+\rho E=0,$$

where *η*_*v*_ denotes the dynamic viscosity. Therefore, we can use Poisson’s equation, i.e. Eq. (5) and the potential distribution of ions to determine **u**.

## Results and Discussions

Finite difference method can be used to solve the PNP equation and the general algorithm for the simulations is given as follow: Given the initial and boundary conditions, and a temperature, under an external electric field, we simulate the flow and force fields of the system, which can be used to update the number density using the Nernst-Planck Equation. The modified number density will then be used to update the velocity and force fields. This forms the basis of an iterative numerical scheme that is repeated until the numerical outcomes converge. We adopt the following non-dimensionalization

11 $$\bar{c}=\frac{c}{C},\,\bar{t}=\frac{Ut}{\ell },\,\overline{{x}_{i}}=\frac{{x}_{i}}{\ell },\,\overline{{F}_{i}}=\frac{\ell {F}_{i}}{m{U}^{2}},\,\overline{{u}_{i}}=\frac{{u}_{i}}{U}$$

where *C*, $\ell$, *x*_*i*_, *u*_*i*_ and *m* denote the initial concentration, the material’s length in the *z* direction, {*x*, *y*, *z*} coordinates, velocities in the {*x*, *y*, *z*} directions and the mass of a single molecule, respectively. Using this non-dimensionalization, Eq. (3) becomes

12 $$\frac{\partial \bar{c}}{\partial \bar{t}}=\frac{1}{Pe}{\nabla }^{2}\bar{c}-\nabla \cdot (\bar{c}\bar{{\bf{u}}})-{\rm{\Upsilon }}\{\bar{{\bf{F}}}\cdot \nabla \bar{c}+\bar{c}\nabla \cdot \bar{{\bf{F}}}\},$$

where $Pe=(U\ell )/D$ denotes the usual Peclet number and ${\rm{\Upsilon }}=(DmU)/({k}_{B}T\ell )$ denotes the comparison between convection and the thermal energy.

Here, we investigate lithium ion transport inside double-layered graphene sheets of the size *L*_*x*_ = *L*_*y*_ = *L*_*z*_ = 6 Å, 8 Å and 10 Å, where *L*_*y*_ denotes the separation of graphene sheets (see Fig. 1). Numerical values for all the constants adopted in this paper are provided in Table 1. As mentioned above, the periodic boundary conditions for *c* in the *x* and *z* directions, the Dirichlet boundary conditions, i.e. *c*(*x*, 0, *z*, *t*) = *c*(*x*, *L*_*y*_, *z*, *t*) = 0 are applied in the *y* direction.

**Table 1:** Numerical values for *A*, *B*, *A*_*i*_, *B*_*i*_ and *S* are given by using the Lorentz-Berthelot mixing rule^34^, where the Lennard-Jones parameters for graphene and lithium ion are extracted from^23^,^24^ and 35, respectively.

Description	Parameter	Value
Attractive constant Li^+1^-Graphene	*A*	5.96 eVÅ^6^
Repulsive constant Li^+1^-Graphene	*B*	2147.68 eVÅ^12^
Repulsive constant Li^+1^-Li^+1^	*B*_*I*_	3.864 eVÅ^12^
Viscosity	*η*_*v*_	0.00018 Pa s
Electric permittivity	*ε*	8.854e-12 Fm^−1^
Number density of graphene	*η*	0.381 Å^−2^

In addition, the numerical value for *η* is given by^23^,^24^.

**Figure 2:**
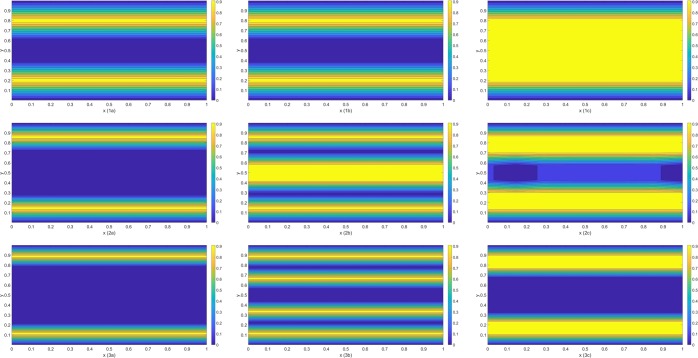
Configurations for lithium ions inside double-layered graphene of various separations, where 1(a–c), 2(a–c), 3(a–c) represent graphene separation of *L*_*y*_ = 6 Å, 8 Å and 10 Å, respectively. In addition, *a*, *b*, *c* denotes the fields of 1e4, 8e5 and 1e8 NC^−1^, respectively.

The spatial grid size is approximately the size of a single lithium ion and the initial uniform concentration of 0.1 is applied throughout the porous cavity. Three external applied electric fields of strength 1e4, 8e5 and 1e8 NC^−1^ are applied for each proposed systems at 300 K. The contour plot of the normalized concentrations in the mid-*z* plane are shown in Fig. 2 after the systems reach stable states.

For the separation of *L*_*y*_ = 6 Å, under the low electric field (see (1a) in Fig. 2), double layers are formed almost simultaneously between graphene sheets. As more ions are introduced into the system, resulting in the oscillation between the double layers and the completely filled cavity (see the double layers in (1b) of Fig. 2 and the completely filled cavity can be represented in (1c)). For even larger electric fields, the cavity is completely filled by ions since the molecular forces induced by the graphene sheets are strong enough to trap the ions (see (1c) in Fig. 2).

**Figure 3:**
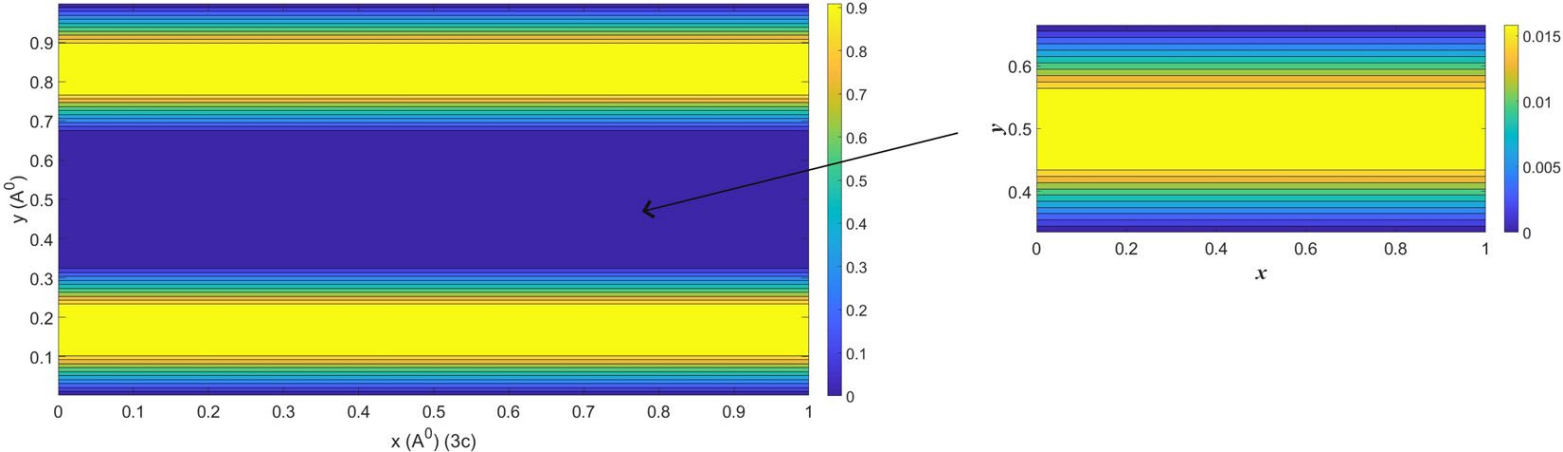
Residual ions in the central cavity of 3(c) in Fig. 2.

**Figure 4:**
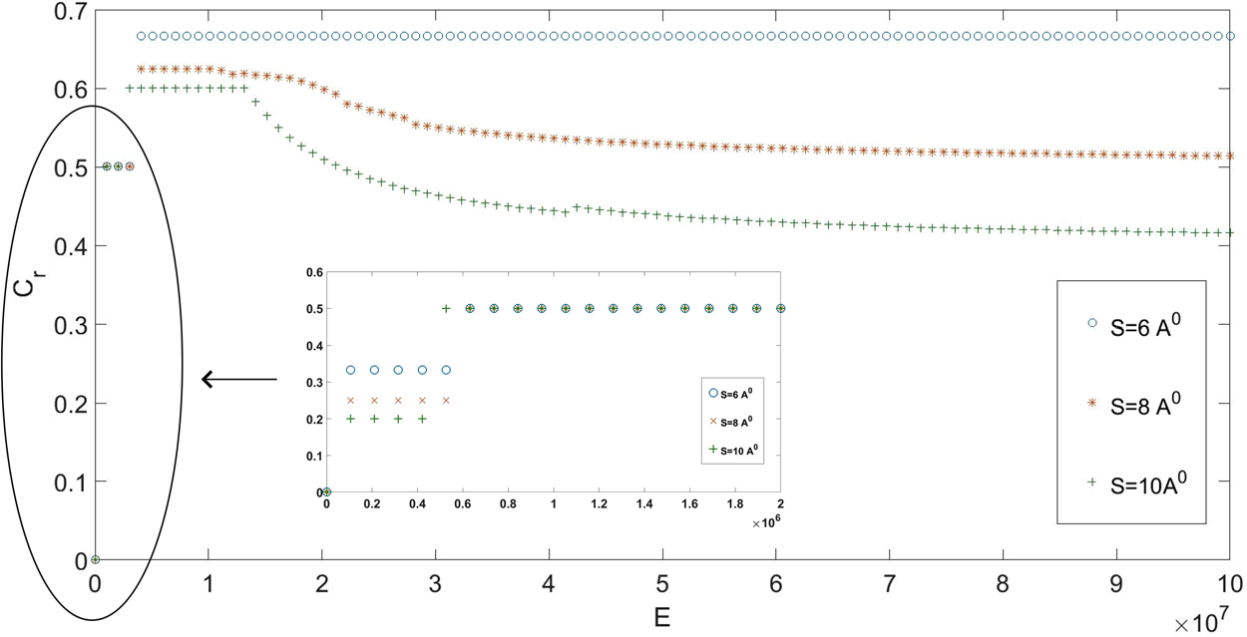
Relative concentration versus external electric fields for the three proposed separations.

On the contrary, when *L*_*y*_ = 10 Å, double layers are formed in the low-field limit (see (3a) in Fig. 2). Multi-layers emerge spontaneously when the strength of electric fields reach a certain threshold (see (3b) in Fig. 2), where the two middle layers oscillate due to the attractive/repulsive forces generated by the layers and the graphene sheets so that the stable ionic structure is primarily maintained by the interactions between layers. However, under large electric fields (see (3c) in Fig. 2), thinner double layers are initially formed. As the repulsive forces between ionic layers cannot be fully counteracted by the weak ion-graphene forces in the central domain, ions are pulled onto the surface of graphene sheets and distinctive multi-layers are unable to form. Instead, the void region forms and few ions are encapsulated in the channel leading to thicker double layers in the vicinity of sheets’ surface. Intriguingly, when we scrutinize the ionic concentrations of the central region, multi-layers still exist but are fluctuating with the lower concentrations (see Fig. 3). This phenomenon is expected to happen for even larger separations under large electric fields but the layers are oscillating with much lower ionic concentrations and are diminished for large separations. The numerical results suggest the transition from the thinner double layers to multi-layers and then into the thicker double layers so that the optimal storage capacity exists for the double-layered graphene of separation 10 Å at 300 K (see Fig. 4 for further details).

To illustrate the transition from *L*_*y*_ = 6 Å to *L*_*y*_ = 10 Å, the results for a separation of 8 Å are shown in 2(a, b, c) of Fig. 2. Results in the low-field limit are similar to those for *L*_*y*_ = 6 Å and 10 Å. While the results for the mid-field limit are similar to those for *L*_*y*_ = 10 Å, significant ionic residuals remain in the middle of channel due to the attraction of some tiny body forces under the large-field limit.

To generalize the results for the above analysis, the effect of the external electric fields on the relative concentration (normalized concentration per unit volume of lithium ions) at 300 K for the three proposed systems is investigated and the numerical results are shown in Fig. 4. We comment that in order to obtain smooth results, only results arising from stable states are used and the relative concentration is calculated by taking the average of those stable results. The relative concentration for all three cases is zero when the external field is zero. When the external field is smaller than 0.6e6 NC^−1^, thinner double layers are formed for all the proposed systems. However, the relative concentration for *L*_*y*_ = 6 Å is lower than that of *L*_*y*_ = 8 Å or 10 Å simply due to the small cavity volume in the case of *L*_*y*_ = 6 Å. Spontaneous emergence of layers occurs around 0.6e6 NC^−1^, where multi-layers occur for *L*_*y*_ = 8 Å and 10 Å whereas the oscillation between the double layers and the completely filled cavity is observed for *L*_*y*_ = 6 Å. For even higher fields, the central cavity of *L*_*y*_ = 6 Å is completely filled with ions whereas thicker double layers form with the fluctuation of lithium ions in between occurring for *L*_*y*_ = 8 and 10 Å.

## Approximate Analytical Solution

Here, we investigate analytically the existence of double layers and multi-layers inside the double layered graphene. In order to perform eigenfunction expansion and solve analytically for *c*, we assume that **F** is constant and **u** to be incompressible so that Eq. (3) admits a neat compact from, which is the usual convection-diffusion equation:

13 $${c}_{t}={\rm{\Delta }}c+{\bf{V}}\cdot \nabla c,$$

where **V** = **u**/*D* + **F**/(*k*_*B*_*T*) so that all the information of force and velocity fields are absorbed in **V**. Due to the symmetry of the present problem, *c* is assumed to be homogeneous in the *x* direction. *c* can be determined using the eigenfunction expansion, where *c*_*t*_ = −*d*_*n*,*m*_*c* for some *d*_*n*,*m*_ ∈ **R** and *n*, *m* ∈ **Z**, and the solution form of *c* is given by $c(t,y,z)=\sum _{n,m}\,{c}_{n,m}\{{T}_{n,m}(t){Y}_{n}(y){Z}_{m}(z)\}$, where *c*_*n*,*m*_ can be determined by an initial condition, and *Y*_*n*_(*y*) and *Z*_*m*_(*z*) are obtained from solving the following ordinary differential equations:

14 $$\begin{array}{ll}\frac{{d}^{2}Y}{d{y}^{2}}+{V}_{y}\frac{dY}{dy}+{k}^{2}Y=0, & {\rm{where}}\,Y(0)=Y({L}_{y})=0\\ \frac{{d}^{2}Z}{d{z}^{2}}+{V}_{z}\frac{dZ}{dz}-\ell Z=0, & {\rm{where}}\,Z\,{\rm{is}}\,{\rm{periodic}}\,{\rm{in}}\,{L}_{{\rm{z}}},\end{array}$$

where *k* and $\ell$ are the separation constants, and *L*_*y*_ and *L*_*z*_ are the separation and the maximum length in the *z* direction, respectively. Due to the boundary conditions for *Y*, *Y* admits the oscillatory solution $Y=\sum _{n}\,{Y}_{n}\,\sin ({\lambda }_{n}y)$, where *λ*_*n*_ = *(nπ*)/*L*_*y*_, *n* ∈ **Z**. In addition, *Z* is periodic with the period *L*_*z*_ so that *Z* can be written as $Z=\sum _{m}\,{Z}_{m}\,\cos ({K}_{m}z)$, where *K*_*m*_ = (2*πm*)/*L*_*z*_, *m* ∈ **Z**. Upon substituting the trial solutions of *Y* and *Z* into Eq. (14), we obtain two algebraic equations for the separation constants in terms of the eigenvalues *λ*_*n*_ and *K*_*m*_.

15 $$\begin{array}{rcl}-{k}_{n}^{2} & = & -{\lambda }_{n}^{2}+{V}_{y}{\lambda }_{n},\\ {\ell }_{m} & = & -{K}_{m}^{2}+{V}_{z}{K}_{m}.\end{array}$$

Furthermore, ${T}_{n,m}(t)={e}^{-{d}_{n,m}t}={e}^{-({k}_{n}^{2}-{\ell }_{m})t}$. If *c* is independent of *t*, we obtain ${k}_{n}^{2}={\ell }_{m}$, and the following equality holds

16 $${V}_{z}{K}_{m}+{V}_{y}{\lambda }_{n}={\lambda }_{n}^{2}+{K}_{m}^{2}.$$

Some observations are provided below for interpreting Eq. (16). Under low external electric fields, without loss of generality, suppose the fundamental mode in *z*, i.e. *m* = 0 and *V*_*z*_ ≈ 0, Eq. (16) reduces to *F*_*y*_/(*k*_*b*_*T*) = (*nπ*)/*L*_*y*_ so that when *F*_*y*_ < < (*πk*_*B*_*T*)/*L*_*y*_, only double layers are formed leading to the classical case of Debye/double layers (This condition will be automatically satisfied when *F*_*y*_ ≈ 0 that automatically happens in micro-channels). Besides, the higher the temperature, the easier for *F*_*y*_ to satisfy such inequality; For small separations and the external filed is not too large, usually ions are oscillating in the *y* direction due to the confinement of the system so that *V*_*y*_ ≈ 0. Therefore, the higher the mode in *n*, the larger *V*_*z*_ is. Hence, the larger electric field is needed to induce multi-layers; Under large external fields, both *V*_*y*_ and *V*_*z*_ come into effect and the results are inconclusive. For small separations, *V*_*y*_ can not be ignored and we expect that modes are divided between *y* and *z*-directions leading to multi-layers; while for large separations, *V*_*y*_ ≈ 0, modes are concentrated in the *z*-direction leading to the classical Debye layers. These observations are consistent with the above numerical results.

## Flow, Conductivity and Thermal Stability

**Figure 5:**
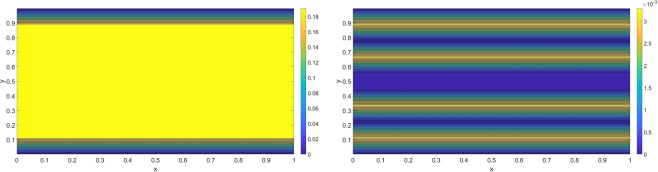
(Left) Velocity and (Right) Conductivity for *L*_*y*_ = 10 Å and *E* = 8e8 NC^−1^.

In the following, we investigate the fluid flow, charge conductivity and the thermal stability for the present problem. Fluid flow is ready to be extracted from Eq. (10) while the conductivity can be computed using the following formula

$${\sigma }_{c}(y)=({\int }_{0}^{{L}_{z}}\,Jdz)/(E{L}_{z}),$$

where *J* denotes the ionic flux inside the graphene sheets^3^. Using *L*_*y*_ = 10 Å as an example, the corresponding contour plots of fluid flow and conductivity when *E* = 8e8 NC^−1^ in the mid-*z* is shown below in Fig. 5.

**Figure 6:**
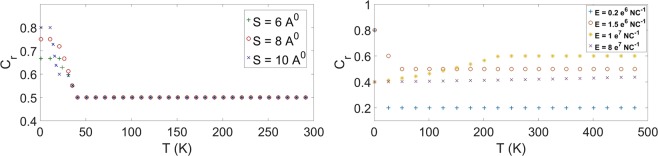
(Left) Relative concentrations for different graphene sheets with *E* = 1.5e6 NC^−1^. (Right) Relative concentrations for four different external electric fields for *L*_*y*_ = 10 Å.

Fluid velocity is almost constant in the central cavity implying that ionic layers are moving almost at the same speed as the velocity driven by the external field, since the applied voltage difference between the graphene dominates the ionic velocity. On the other hand, conductivity correlates exactly with the position of the multi-layers revealing that the higher charge conductivity occurs in the regime of ionic layers. Finally, we investigate the thermal stability for the three proposed cases when *E* = 1.5e6 NC^−1^, where stable multi-layers are formed for *L*_*y*_ = 8 Å and 10 Å at 300 K (see Fig. 4). The relative concentrations versus temperatures are shown in Fig. 6.

We comment that the relative concentrations for all the proposed systems fall sharply when *T* < 50K and stay almost constant for normal daily operational temperatures. We suspect that the strength of external field might also cause the sharp drop. To test such a hypothesis, both field and temperature effects on the relative concentrations are investigated. We fix *L*_*y*_ = 10 Å and propose four distinctive field strengths, i.e. 0.2e6, 1.5e6, 1e7 and 8e7 NC^−1^, where the effect of fields on the ionic concentration for different temperatures is shown in Fig. 6. The relative concentrations decline for *E* = 0.2e6 and 1.5e6 NC^−1^ but decline less sensitively for *E* = 1.5e6 NC^−1^ in comparison to that for *E* = 0.2e6 NC^−1^ due to the fact that more ions have intruded into the system and the more stable multi-layers are formed for the case of *E* = 1.5e6 NC^−1^. On the contrary, a surprising rise of relative concentrations happens under the large-field case such as *E* = 1e7 and 8e7 NC^−1^ when the temperature increases but eventually reaches a plateau. We suspect that such systems saturate under the large-field limit and the extra increase in thermal energies raises the chance of lithium ions anchoring to the surface of graphene sheets. In addition, the more rapid rise for *E* = 1e7 NC^−1^ in comparison to *E* = 8e7 NC^−1^ due to the more stable and thinker double layers are formed for *E* = 8e7 NC^−1^. In conclusion, the thermal stability of relative concentrations appears to correlate with the stability of systems.

We comment that while double Debye layers always occur in ionic channels with sufficiently large separation^1^, multi-layers have been observed in several works^3,5^. However, prior to this work, the transition from double layers to multi-layers and vice versa had not been studied. We have performed a detailed parametric study of this transition and presented a simple theory that captures many of the key features of the dynamics. These issues were not address in^5^. We end by noting that multi-layers are found to accommodate significantly more ions than double layers and hence have the potential to be of significant practical importance in applications such as lithium-ion batteries in which high-density charge storage is a major issue.

## Conclusion

Layer analysis is performed for the three proposed double-layered graphene sheets. For graphene sheets with extremely small separations, only double layers can be formed in the small and mid-range electric fields due to the repulsive force between ions. However, due to the strength of molecular forces inside the cavity, the cavity is completely filled with lithium ions under the large-field limit. On the other hand, the transition of the thinner double layers into the multi-layers and then into the thicker double layers (with fluctuating ions in between) occurs for relatively large separations. Only double layers form under large electric fields as the weak molecular force are not able to maintain the structural stability of multi-layers. The relative concentrations versus the applied electric fields are reported, and the fluid flow and charge conductivity are also analyzed. Finally, the thermal stability is discussed and we discover that the relative concentrations decline under the low- and mid-field limits, whereas the relative concentrations raise over the temperatures under the large-field limit as more ions adhere with the graphene sheets due to thermal fluctuation. The thermal stability of the relative concentration is thus closely related to the stability of the system. The present results bridge the gap between the classical theory for Debye layers and the multi-layers that occur at the nanoscale.
